# Functional miscibility and thermomechanical properties enhancement of substituted phthalic acetylated modified chitin filler in biopolymer composite

**DOI:** 10.1098/rsos.211411

**Published:** 2022-06-01

**Authors:** N. G. Olaiya, O. S. Obaseki, Gaber A. M. Mersal, Mohamed M. Ibrahim, Mahmoud M. Hessien, Olaiya Funmilayo Grace, Asif Afzal, Taslima Khanam, Ahmad Rashedi

**Affiliations:** ^1^ Department of Industrial and Production Engineering, Federal University of Technology Akure, PMB 704, Ondo state, Nigeria; ^2^ Department of Physical Sciences, Landmark University, PMB 1001, Omu-Aran, Kwara State, Nigeria; ^3^ Department of Chemistry, College of Science, Taif University, P.O. Box 11099, Taif 21944, Saudi Arabia; ^4^ School of Industrial Technology, Universiti Sains Malaysia, 11800 Penang, Malaysia; ^5^ Department of Mechanical Engineering, School of Technology, Glocal University, Delhi-Yamunotri, Marg, SH-57, Mirzapur pole, Saharanpur District, Uttar Pradesh 247121, India; ^6^ University Centre for Research and Development, Department of Mechanical Engineering, Chandigarh University, Gharuan Mohali, Punjab, India; ^7^ College of Engineering, I.T. and Environment, Charles Darwin University, Ellengowan Drive, Casuarina, NT 0810, Australia

**Keywords:** miscibility, characterization, thermomechanical, chitin, modification, substitution

## Abstract

The miscibility between hydrophobic and hydrophilic biopolymers has been of significant challenge. This study used a novel simplified chitin modification method to produce phthalic chitin using phthalic anhydride in a substitution reaction. The FT-IR functional group analysis was used to confirm the substitution reaction. The modified chitin was used as compatibilizer in polylactic acid (PLA)/starch biocomposite to enhance its properties. The biocomposite was prepared using melt extrusion and compression moulding technique. The biocomposite's morphological, thermomechanical and water absorption properties were characterized using scanning electron microscope, tensile test, dynamic mechanical analysis, thermogravimetry analysis, differential scanning calorimetry, thickness swelling and water absorption test. The FT-IR study shows a successful substitution reaction of the amine hydrogen ion present in the chitin as opposed to substituting the hydrogen ion in the hydroxide group. The tensile and impact properties of biocomposite incorporated with modified chitin showed better results compared with other samples. The SEM images showed uniform miscibility of the modified biocomposite. The dynamic mechanical analysis showed improved modulus value with the incorporation of modified chitin. The thermal properties showed improved thermal stability of the modified biocomposite. Furthermore, the percentage of water absorbed by biocomposite with modified chitin is reduced compared with the PLA/starch biocomposite. The produced biodegradable ternary blend can be used as a substitute for plastics in industrial applications.

## 1. Introduction

Biodegradable polymers, or polymers derived from biological sources, are a viable substitute for many petrochemical polymers. They are, however, challenging to work with because property variance is always source dependent [1,2]. Polylactic acid (PLA) is a polymer with characteristics similar to polyethylene terephthalate (PET) and has flexibility akin to polypropylene (PP). Because of its ability to be stress crystallized, thermally crystallized, impact modified, filled, copolymerized and processed in most polymer processing equipment, it may end up being the polymer with the most extensive range of applications [3,4]. PLA also offers good organoleptic properties, making it appropriate for food contact and related packaging applications. PLA is also used in three-dimensional printing because of its low flow temperature, which allows it to flow through a three-dimensional printer's laser tip [5,6]. PLA also emits H_2_O and CO_2_, but its decomposition is not harmful to humans or the environment. PLA becomes an environmentally beneficial biomaterial as a result.

PLA undergoes a lengthy hydrolysis process that can take years to complete. It is extremely brittle, resulting in poor strength and a breaking point of less than 10% elongation. PLA is also hydrophobic and has poor gas barrier characteristics [7,8]. PLA is required because of the wide range of uses, and many researchers believe that PLA may be improved and treated to address the disadvantages. PLA can be bulk-modified, surface-modified or physically altered. This makes the molecular chain more flexible and crystalline [6,9].

Starch is the most cost-effective biodegradable substance used in various non-food applications such as papermaking, cardboard, textile sizing, thickening, adhesives agents and kitchenware and film raw materials. Furthermore, starch has the advantage of having quick-dissolving qualities in polymers, and starch-based polymers are often used in food packaging, where the plastic can deteriorate with the food. However, a peculiar physical and chemical reaction occurs [10]. Because of their poor water tolerance and weak mechanical capabilities under moist circumstances, starch-based materials have been limited thus far. As a result, starch is commonly combined with different polymers to broaden its application [11,12]. The morphology and characteristics can be adjusted by altering the components in the mix and manipulating their interaction with starch. As a result, building a new generation of biodegradable starch-based blends requires a good understanding of the mechanism that controls the process-structure–function link in the blend.

PLA has been blended with starch to achieve higher strength because of the semi-crystalline nature of starch. However, the agglomeration challenge between PLA and starch has resulted in limited application of the blend. Several techniques have been applied to improve the PLA/starch blend [13], classified numerous tactics used to improve PLA and starch blend miscibility. Plasticization, softening, toughening and compatibilization were stated in their review on PLA/starch blend state of the art. The strategies are not mutually exclusive, according to the research, and can be used together in a single blend, allowing for a wide range of approaches to be used in the PLA/starch blend. The review suggested a straightforward, bio-based approach for improving PLA/starch miscibility with outstanding mechanical properties suited for a wide range of applications may be developed. This is the subject of this study.

Chitin is a white, nitrogenous, inelastic polymer found in various places, including crustacean exoskeletons, fungal cell walls, diatoms, mushrooms, worms, arthropods, insects, crawfish, shrimp and crabs, excrement being the most common sources [14]. Chitin, after cellulose, is the most abundant and essential natural polysaccharide on the planet. It comprises N-acetyl glucosamine and (14)-linked 2-acetamido-2-deoxy-D-glucose [15,16]. Chitin, N-acetyl chitin, monoacetyl chitin, di-butyryl chitin, chitin acetate and other chitin derivatives are among them. Chitin, a linear polymer of (14)-linked 2-amino-2-deoxy-D-glucopyranose, is a direct derivative of chitin that N-deacetylation easily generates to different degrees characterized by the degree of deacetylation. As a result, this is an N-acetyl glucosamine/glucosamine copolymer [15,17]. Modified chitin was created by modifying chitin to have both hydrophilic and hydrophobic characteristics. The modified chitin was used as compatibilizer in the PLA/starch blend to enhance its blend. The morphology, thermomechanical properties and water absorption of the blend were evaluated for improved properties. The modification used in this study was adapted and simplified from previous studies. As opposed to substitution reaction taking place in the hydroxide functional group of chitin; in this study, the hydrogen ion of the amine group was substituted, which enhanced the compatibilizer properties of chitin. The use of modified chitin as a compatibilizer biopolymer composite blend has not been researched.

## 2. Material and methods

### 2.1. Material

The materials are polylactic acid, starch and chitin. The PLA was used as the matrix of the polymer blend biocomposite. Nature Works (Minnetonka, MN, USA) supplied commercial quality poly(lactic acid) (4032D) in pellet form. PLA (4032D) was used because it possesses outstanding oil and grease resistance, excellent twist and machinability properties. It has a glass transition temperature of 60–65°C, a melting point of 155–170°C, a tensile strength of 53.5 MPa, and a modulus of 3500 MPa. PLA (4032D) has a molecular weight of 100 000 g mole^−1^, a specific gravity of 1.24, and a melt flow rate (MFR) of 0.78 g min^−1^ (210°C, 2.16 kg). Lifeline Nutrition, Chicago, IL, USA, provided commercially available chitin deacetylated to 90%. Sigma Aldrich provided commercial high-amylose corn starch (Sigma Aldrich, Modderfontein, South Africa).

### 2.2. Preparation of modified chitin and biocomposite

The chitin was mixed with phthalic anhydride in dried dimethylformamide (DMF) and heated to obtain phthayl chitin (PHCS), used to make modified chitin. It came in the form of a yellow powdered substance. In 100 ml of acetic acid, the chitin powder was dissolved. Phthalic anhydride in methanol was poured into the chitin solution and heated at 50°C with stirring for 8 h. The obtained colloid mixture was precipitated with NaHCO_3_ for 1 h with continuous stirring to form a semi-solid gel. The semi-solid gel was removed and rinsed in distilled water until it reached a pH of 7. The gel precipitate was then dried by freezing and ground to powder. For testing and biocomposite manufacture, the modified chitin powder was stored in an air-tight bag. FT-IR functional group analysis of chitin and modified chitin was performed with FT-IR EFTEM Libra, Carl Zeiss, UK. The samples were mixed with potassium bromide (KBr) in ratio 1 : 10 and pressed into pellet.

Table 1 shows the percentage composition variation between PLA, chitin, modified chitin (Mchitin) and starch [3,18,19].

**Table 1.** Composition variation of the biocomposite.

sample	PLA (wt%)	starch (wt%)	chitin (wt%)	modified chitin (wt%)
neat PLA	100	0	0	0
PLA/starch	90	10	0	0
PLA/chitin/starch	90	5	5	0
PLA/Mchitin/starch	90	5	0	5

Dry air generator Luxor 50 (Motan, Überlingen, Germany) was used to dry the PLA pellets at 60°C for 4 h. Using a Thermo electronic rheomixer 03, powdered chitin, Mchitin, and starch with various compositions were combined with a constant percentage of PLA fluctuation (Thermo Fisher Scientific, Waltham, USA). A Process 11 twin-screw extruder was used to mix and extrude the mixture (Thermo Fisher Scientific, Waltham USA). The extruding temperature ranges from 120°C to 200°C, and the product is subsequently quenched in room temperature in water. The sample filaments were pelletized in a Thermo Scientific pelletizer (Thermo Fisher Scientific, Waltham, USA) and pressed into characterization test forms in a Carver press compression moulding machine (Carver, Wabash, USA) at 170°C for 15 min at 10 MPa pressure. The obtained test samples were air-cooled in atmospheric air and stored in lock bags.

### 2.3. Characterization of the biocomposite

The neat PLA and the biocomposite were characterized with dynamic mechanical analysis (DMA), thermogravimetry analysis (TGA), derivative thermogravimetry (DTG), tensile test and water absorption analysis at ASTM standard. The morphological properties were studied using scanning electron microscopy (SEM). This work employed dynamic mechanical analysis to assess the miscibility of polymeric blend systems and analyse polymers' mechanical and structural properties. Furthermore, the dynamic mechanical analysis depicts the composites' mechanical properties at various temperatures. A PerkinElmer dynamic mechanical analyser (DMA 8000) was used (PerkinElmer Inc., OH, USA). At a frequency of 1 Hz and a temperature range of 50°C to 150°C, samples were manufactured according to the ASTM D4065 standard for polymer composites. The thermogravimetric study was used to investigate the composite's thermal deterioration behaviour. T.A. Instruments (PerkinElmer Inc., OH, USA) provided the PerkinElmer TG-IR-GCMS Interface Q500. T.A. universal analysis programme was used to analyse the results (T.A. instruments, Lukens Drive New Castle, USA). The thermogravimetric test was carried out according to ASTM E1131, with sample volumes ranging from 10 to 21 mg being heated at a rate of 10°C min^−1^ from room temperature to 600°C in an air bath. The neat PLA and biocomposite differential scanning calorimetry (DSC) was analysed with DSC model 6 PerkinElmer (Schwerzenbach, Switzerland) using ASTM 3418 to observe the heat flow properties change with temperature. The transition temperatures were obtained for a sample of 6 to 7 mg.

An Instron universal testing machine (model 5966) (Instron, Norwood, USA) was used to perform the tensile test, run at a constant crosshead speed of 2 mm s^−1^ with a 30 kN load. The composites specimen was created and submitted to the American standard for a material test with cross-sectional dimensions of 5 by 10 mm and a gauge length of 30 mm. The ASTM D3039 tensile test technique was used with standard dimensions. The impact strength was obtained using Ceast Resil Impactor 7181 (Akron, OH, USA), a hammer. The purpose of the test is to determine how much energy is absorbed throughout the breaking process. Scaffolds are subjected to impact loading. Hence this is required. The ASTM D256 standard specimen with dimensions of 10 × 10 mm by 50 mm was used. Before being placed in the impactor, the samples were notched in the middle. The microstructure of the various samples (parts) of the experiments was thoroughly investigated using scanning electron microscopy (SEM). SEM with both secondary electron (SE) and backscattered electron (BSE) signals were used to examine the microstructure of carbon-coated materials. The SEM was operated at an accelerating voltage of 2.00 kV secondary electron imaging (SEI). The material samples were immersed in the distilled water for 24 h. The initial and final mass of the samples were used to calculate the absorption rate. The ASTM test method D570-81 was used to determine the water absorption.

## 3. Results and discussion

**Figure 1.**
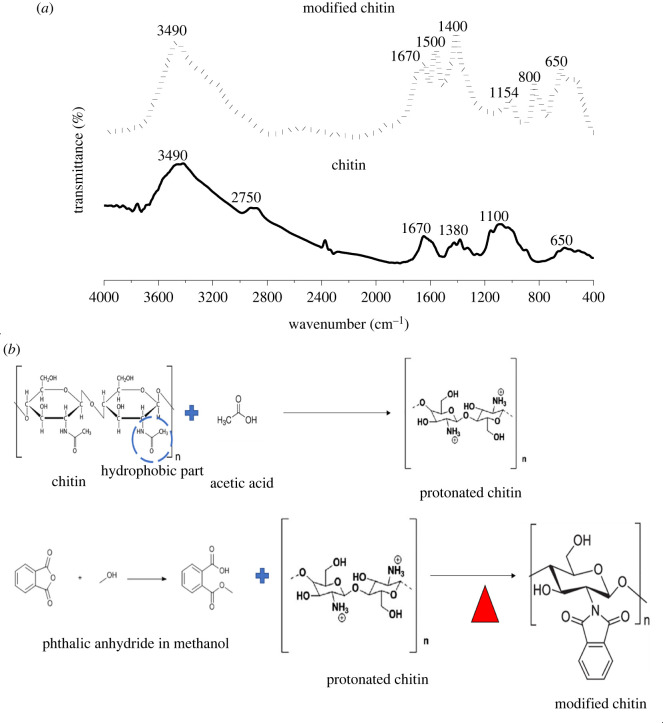
(*a*) FT-IR analysis of chitin and modified chitin (*b*) schematic chemical reaction showing the preparation of modified chitin.

Figure 1 presents the FT-IR functional group analysis of chitin modification. Both unmodified and modified chitin displayed a band between 3100 and 3500 cm^−1^, indicating presence of water, -OH bond and NH stretches, respectively. On the chitin graph, a minor peak was found between 2810 and 2920 cm^−1^, indicating aldehyde -C-H stretches which were not found in modified chitin, probably because of the substitution reaction. Both modified chitin and unmodified chitin showed C = C alkene stretch, C = O at 1670 cm^−1^, and sp^2^ C-H bends at 1380 and 1400 cm^−1^. However, C = C aromatic stretch at 1500 cm^−1^ in the modified chitin indicates phthalic aromatic alkyl substitution, which is not present in the unmodified chitin. Also, 1100 and 1154 cm^−1^ C-O-C bridge was present in both. The presence of 800 cm^−1^ bands in the modified chitin indicated an alkene C-H bend [20]. An increase in 650 cm^−1^ bands indicated a C-H bond, which indicated an alkyl group. A classic sign of alkyl group attachment (substitution) to the chitin chain is the appearance or alteration of new peaks bands 1500 and 800 cm^−1^ [21]. The alkyl group from the phthalic anhydride was attached to the amine group substitution for the hydrogen ion [22]. That is, during the reaction, the hydrogen ions of -NH_2_ were replaced.

**Figure 2.**
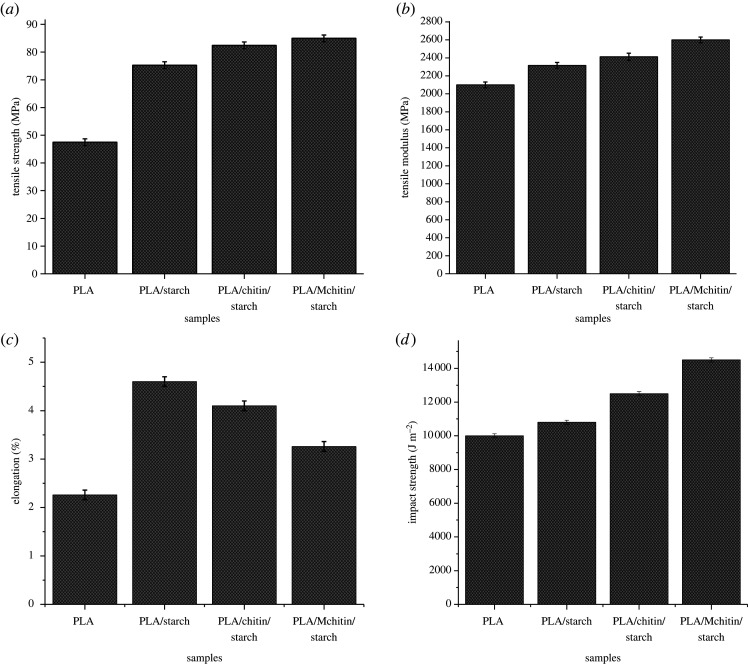
(*a*) Tensile strength (*b*) tensile modulus (*c*) elongation (*d*) impact properties of neat PLA and biocomposites.

The tensile properties of the biocomposites are shown in figure 2. The biocomposite's tensile strength (figure 2*a*) increased more than the neat PLA (53.5 MPa). The highest tensile strength for the biocomposite was observed with PLA/Mchitin/starch with 87.5 MPa, followed by PLA/chitin/starch with chitin and starch filler. The result showed that the biocomposite's tensile strength is enhanced with chitin and starch, but both filler's effectiveness justifies blending PLA with chitin and starch. The increase in tensile strength is probably due to the interaction between the three polymeric materials [23]. The tensile strength result is probably an indication of good miscibility [24]. The previous report showed that the addition of starch in the PLA matrix enhanced its strength, and PLA filled with chitin [18,25,26]. However, starch was reported with agglomeration in PLA due to differences in nature. PLA is hydrophobic, and starch is hydrophilic. Furthermore, chitin filled in PLA was reported to show better miscibility properties than PLA filled with starch. However, the use of both chitin filled in PLA/starch showed better properties probably due to its hydrophobic properties, enhancing its miscibility with PLA and the presence of hydroxide functional group in chitin structure, enhancing its miscibility with starch. This dual nature (hydrophobic and hydrophilic) was enhanced by modifying chitin with amine group hydrogen replacement with phthalic alkyl chain, resulting in better PLA/Mchitin/starch results.

The tensile modulus of neat PLA and biocomposite is presented in figure 2*b*. The tensile modulus value of neat PLA is 3500 MPa. Generally, the modulus of the biocomposite is significantly higher than those of the neat PLA. Similar to the tensile strength results, the modulus of PLA/Mchitin/starch shows the highest strength while that of PLA/chitin/starch is next to it. The lowest among the biocomposites was obtained with PLA/starch, which has the highest starch percentage, due to the difference in nature of PLA and starch. The tensile modulus of biocomposites increased by more than 20% for PLA/Mchitin/starch compared with the neat PLA. This is quite a significant increase which showed the compatibility between PLA, Mchitin and starch.

The elongation properties of the neat PLA and biocomposite are presented in figure 2*c*. The elongation of the neat PLA shows the lowest due to its brittleness. The elongation value of the biocomposite showed significant improvement compared with the neat PLA. Also, the elongation values were observed to increase with both starch and chitin. The highest elongation value was obtained for PLA/starch due to its film-forming ability. The elongation is seen to reduce with the inclusion of chitin in the biocomposite but is still higher than that of neat PLA. This showed that the addition of chitin and starch enhanced the elongation properties of PLA, which means the brittle nature of PLA was reduced. This is probably due to the film-forming property of chitin and starch [27]. This showed that the addition of filler also enhanced the elongation of the biocomposite. The literature reported the elongation trend with the PLA blend with chitin and starch [5,28].

The impact strength of the material was also carried out to show its resilience property. The graph of energy and resilience against samples is shown in figure 2*d*. The impact strength of a material is a measure of its resistance to a sudden force. The impact strength follows a trend similar to tensile strength and modulus, with starch and chitin percentages increasing more than neat PLA. The low impact strength of neat PLA probably means that it has a brittle nature. Notably, the graph shows PLA/Mchitin/starch having the highest impact strength, which shows a good toughening percentage. The graphs show a steady increase in the impact of energy, having the highest impact value at 14 200 J m^−2^. The effect of chitin and starch on the impact strength of the samples is more significant, as shown with PLA/chitin/starch compared with PLA/starch. This results from the brittle nature of the starch-filled biocomposite, which results in its low value, as reported previously [29,30].

**Figure 3.**
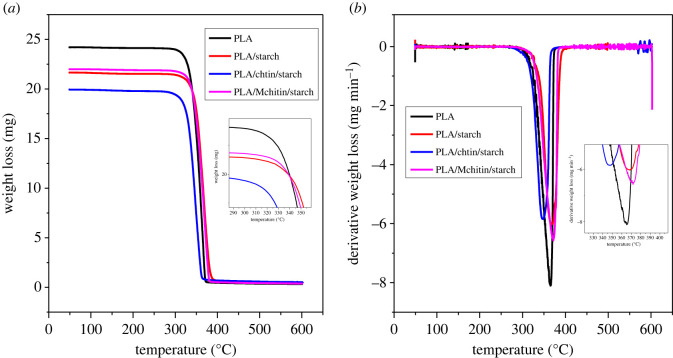
(*a*) Thermogravimetry analysis (TGA), (*b*) derivative thermogravimetry analysis (DTG) properties of neat PLA and biocomposites.

Figure 3 shows the results of thermogravimetry analysis (TGA) and derivative thermogravimetry (DTG) analysis of the plain PLA and biocomposite. The graph depicts the weight loss % as a function of temperature change, which measures the composite's heat stability. The TGA curve (figure 3*a*) revealed a single deterioration curve for neat PLA and the biocomposites. The single curve deterioration is most likely a favourable indicator of the polymeric blends' good miscibility. The onset temperatures are 303.11°C, 290.8°C, 289.75°C and 291.45°C for plain PLA, PLA/starch, PLA/chitin/starch and PLA/Mchitin/starch, respectively. The onset temperature of the biocomposite is generally reduced with starch content due to its biodegradable nature. The onset temperature is further reduced with chitin and Mchitin, enhancing degradation [31,32]. The onset temperature values indicated that addition of chitin and starch affects the degradation process. It is expected that the thermal degradation of PLA would be enhanced due to the addition of natural polymer, which has also been reported in previous studies [31].

Furthermore, the DTG curve (figure 3*b*) peak temperature values for neat PLA, PLA/starch, PLA/chitin/starch, and PLA/Mchitin/starch are 366.67°C, 367.10°C, 358.58°C and 370.21°C respectively. These results show that the addition of starch has a minor effect on the DTG peak temperature compared with the plain PLA. However, combining starch and chitin (PLA/chitin/starch) resulted in a considerable reduction in DTG peak temperature, which improves the biocomposite's thermal degrading capabilities. Also, Mchitin loading increased the DTG, which is probably due to miscibility enhancement. The reduction in the DTG peak temperatures is corroborated with the residue percentage. The percentage weight losses for neat PLA, PLA/starch, PLA/chitin/starch and PLA/Mchitin/starch are 87.08%, 92.04%, 90.40% and 95.04%, respectively. The percentage of weight loss is increased due to the natural biopolymer filler degradation enhancement of neat PLA. The values of the residue for neat PLA, PLA/starch, PLA/chitin/starch and PLA/Mchitin/starch residue are 10.54%, 6.24%, 7.78% and 4.92%, respectively. The difference in weight loss and residue is due to the volatile content present in the biocomposite [18].

**Figure 4.**
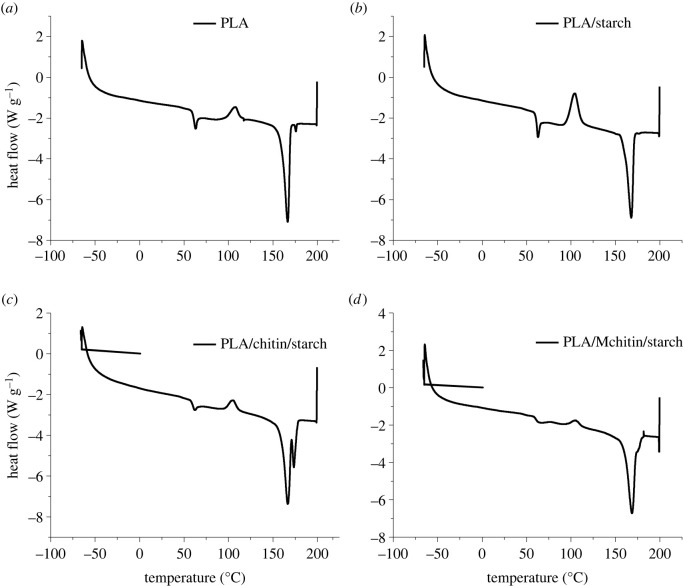
Differential scanning calorimetry of PLA, PLA/starch, PLA/chitin/starch and PLA/Mchitin/starch biocomposites.

The differential scanning calorimetry (DSC) result is shown in figure 4 and table 2 for neat PLA and biocomposites. The glass transition temperature was observed to reduce with starch and chitin, which shows enhanced degradation properties [33]. However, the glass transition temperature of the biocomposite with modified chitin has a slightly higher value, probably due to the improved interfacial interactions as a result of the modification. Also, the crystallization temperature was generally reduced compared with the neat PLA, but the biocomposite with modified chitin had the highest temperature. The melting temperature of the neat PLA and biocomposite shows no specific trend. The result corroborates the thermogravimetry analysis, which shows that PLA degradation was enhanced with chitin and starch. The enthalpy of the neat PLA and biocomposite is very low, which shows that it cannot retain heat for a long time and is an indication of an amorphous material. Previous studies also show a similar trend of degradation enhancement of biopolymer filled in polylactic acid [24,34]. This was attributed to the degradation properties of most natural biopolymers. Polylactic acid is more amorphous. This accounts for the low transition temperature of its biocomposite [8].

**Table 2.** Differential scanning calorimetry transition temperatures values of neat PLA and biocomposites.

sample	glass temperature (°C)	crystallization temperature (°C)	melting temperature (°C)
PLA	64.7	107.1	167.2
PLA/starch	63.5	104.4	168.1
PLA/chitin/starch	62.5	104.7	167.2
PLA/Mchitin/starch	65.9	105.6	168.3

**Figure 5.**
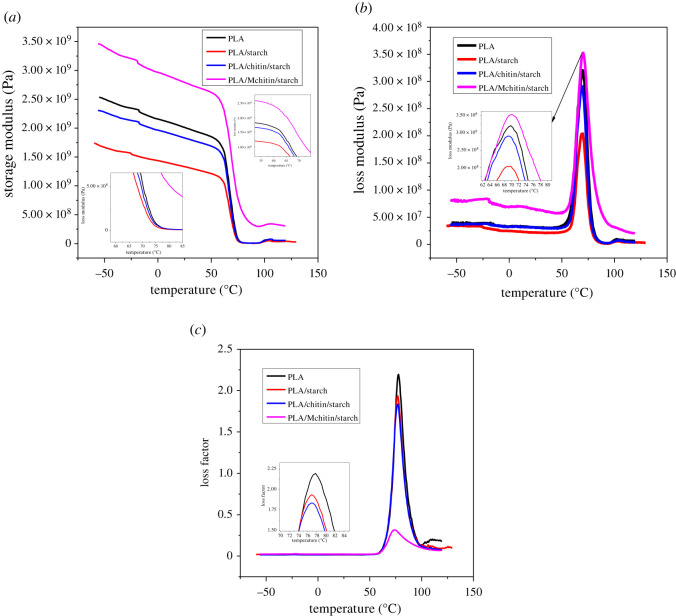
Dynamic mechanical analysis (DMA) (*a*) storage modulus, (*b*) loss modulus and (*c*) loss factor of neat PLA and biocomposites.

The variation of storage modulus (*E*′), loss modulus (*E"*) and loss factor (tan *δ*) of the biocomposites with temperature under 1 Hz are shown in figure 5. The storage modulus (*E*′) value of the neat PLA and biocomposite is presented in figure 5*a*. The storage modulus graph shows the thermomechanical behaviour of the biocomposite from the glassy region to the rubbery region [35]. The addition of starch loading to PLA significantly affects the *E*′ value of the biocomposites. The *E*′ reduced with the addition of starch, as can be seen in figure 5*a*. This meant that the damping properties of composites reduced, and they became more brittle with the increase in starch content. Compared with PLA/starch, the storage modulus increased with chitin (PLA/chitin/starch) but was slightly lower than neat PLA. However, the highest storage modulus was observed with PLA/Mchitin/starch, probably enhanced miscibility. The storage modulus shows the stiffness properties of the material with temperature change [36]. The graph shows that the material's storage modulus is maintained above 72°C, which shows the material is suitable for use below this temperature because the mechanical reduced drastically after the temperature. The high modulus value of PLA/Mchitin/starch showed significant enhancement compared with other biocomposites.

The loss (viscous) modulus graph (figure 5*b*) shows the ability of the biocomposite to store energy from the glassy region to the rubbery region. The loss modulus lowest value was observed with PLA/starch followed with PLA/chitin/starch and neat PLA. The loss modulus value of PLA/Mchitin/starch showed the highest value, similar to the storage modulus graph. The peak of the loss modulus was observed at 72°C, above which the modulus value decreases. The loss modulus value *E"* increased up to 3600 GPa with a Mchitin sample, while the modulus value was observed to reduce with chitin content. This shows the modification process's significance, which enhances miscibility in PLA/Mchitin/starch biocomposites. Generally, the loss modulus is high because of the viscoelastic properties of biopolymer. The loss modulus increased in value until 72°C, which signifies the glass transition into the rubbery region. The modulus begins to reduce at a higher rate until it turns into a viscous liquid. Above this temperature, the material possesses low internal bonding due to viscosity. This is similar to the storage modulus reduction, which shows a uniform line that signifies that the polymer blend is homogeneously mixed [18]. The PLA/Mchitin/starch biocomposite shows enhanced stiffness compared with neat PLA. This means that Mchitin has good miscibility with polylactic acid and starch in the biocomposite; therefore, the energy storage modulus also increased [18].

Figure 5*c* presents the corresponding loss factor (tan *δ*) curves of the biocomposites. This shows the damping properties of the biocomposite. The chitin-based (PLA/chitin/starch) biocomposites (PLA/starch) showed the lowest tan *δ* peak and temperature, attributed to poor interactions between the reinforcement and the matrix [37]. There was a pronounced peak of the neat PLA above the biocomposites due to its pure nature. However, the peak temperature of PLA/starch and PLA/Mchitin is slightly lower than neat PLA. The peak of the loss factor represents the transition temperature of the material [37]. This shows that the glass transition temperature of the biocomposite is lower, which signifies enhanced degradation properties. This is expected because starch and chitin are biodegradable polymers [36].

**Figure 6.**
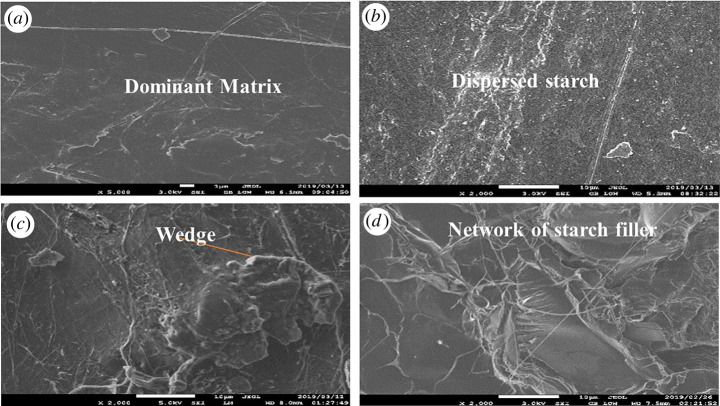
Morphological properties of (*a*) neat PLA (*b*) PLA/starch (*c*) PLA/chitin/starch (*d*) PLA/Mchitin/starch.

The material samples were examined under scanning electron microscopy to determine the influence of the composition variation on the morphological properties. Figure 6*a–d* shows the SEM images of neat PLA and biocomposites. Homogeneous surface morphologies were observed for the samples with neat PLA. However, the surface characteristic of the image changed with the addition of starch (figure 6*b*) with characteristics of white starch patches. The PLA/starch SEM images show flakes of starch. This image shows that the starch was not miscible with the PLA matrix due to differences in their nature. This is expected due to the difference in the nature of the two polymers. Polylactic acid is hydrophobic, while starch is hydrophilic. This means that starch immiscibility is dependent on the presence of hydroxyl ions. Starch has been reported to mix with water-loving polymers such as seaweed. However, PLA is highly repellent to water, resulting in immiscibility and probably the features experienced in the SEM images [25]. The lack of visible void in the SEM images may be due to the reduced particle sizes of PLA and starch, which improved their miscibility [25]. These images show that the miscibility between PLA and starch is mainly due to interfacial interaction due to the large surface area due to the reduced particle size difference [25]. The samples containing chitin and starch (figure 6*c*) had a rougher surface than the PLA/starch.

However, distinct white patches observed with PLA/starch had disappeared. This can be attributed to the inclusion of chitin in the blend with mixes with the starch filler. Chitin has been reported to have miscibility with starch due to the hydroxyl group in its structure. However, chitin is still hydrophilic, which also makes it possible for mixing with PLA. The image was observed to have wedges of the filler in the matrix. The surface of the samples with the Mchitin and starch showed crater-like dips on its surface with a uniform colour mix and network of lines (figure 6*d*). The image showed a rough surface, but the uniformity in its colour shows the uniform mix.

**Figure 7.**
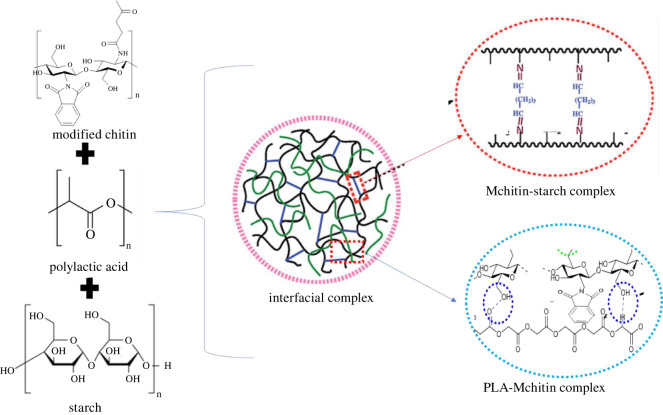
Schematic chemical reaction showing the possible complex interfacial interaction between PLA, Mchitin and starch.

The nature of the polymer blends can be used to explain the miscibility as shown in figure 7. Because PLA is hydrophobic and starch is hydrophilic, they are thermodynamically incompatible [38]. The miscibility of the mix, on the other hand, can be explained by the evaluation reports of [19] and [39]. It was reported that by employing a material that is highly compatible with PLA and starch, the adhesive qualities of PLA/starch could be improved [13]. Based on chitin good adherence to PLA [40,41] and starch [42–44], it was modified to form ‘Mchitin’ to suit this purpose. As a result, the Mchitin in this mix acts as an interfacial compatibilizer, bridging interfacial transitions between PLA and starch [13,45].

**Figure 8.**
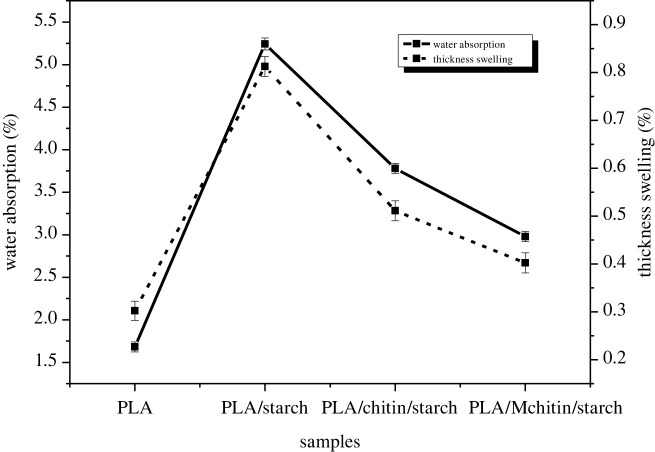
Water absorption and thickness swelling properties of neat PLA and biocomposites.

Water absorption is a critical feature of a biodegradable polymer composite that assesses the material's resistance to water. Polylactic acid and chitin are hydrophobic, while starch is a hydrophilic material [18,41]. As a result, starch, a hydrophilic polysaccharide, is responsible for a portion of water absorption. Figure 8 shows the proportion of water absorption for several samples after 24 h. After 24 h of immersion in distilled water, all samples show a downward trend with varying values. The biocomposite's low water absorption rating indicates that it is highly hydrophobic [41]. Therefore, it can be deduced that the water absorption rate increases with an increase in the percentage of starch but is reduced with PLA and chitin. The sample with the lowest value is neat PLA because of its hydrophobic nature, and the highest is PLA/starch due to its starch content.

The capacity of water molecules to enter through the starch network causes water absorption. The nature of the polymer, which has high interaction with water, is blamed for water diffusion in starch. The low water absorption of PLA and chitin has also been reported by Nasrin *et al.* [41] in their PLA/chitin biocomposite report. The percentage of starch increased the composite water absorption in this investigation. Starch absorbs water more efficiently than chitin due to the hydrogen interaction between the water molecule and the starch in the polymer composite. This is why PLA/starch biocomposite (with the highest starch component) absorbs the most water. The hygroscopic property of starch also contributes to its water absorption [18]. Because the composite absorbs water slowly and degrades slowly, the results revealed that the composite is suited for water-resistant applications such as packaging.

The thickness swelling of neat PLA, PLA/starch, PLA/chitin/starch and PLA/Mchitin/starch biocomposites after immersion in water for 24 h is plotted in figure 8. The thickness of the PLA/starch, PLA/chitin/starch and PLA/Mchitin/starch increased compared with the neat PLA. The neat PLA has the lowest thickness of swelling. PLA/starch biocomposite increase is compared with neat PLA and has the highest value [10]. However, the incorporation of chitin in the biocomposites reduced swelling values. The reduction in the chitin and Mchitin biocomposite's swelling value is probably due to the hydrophobic part of chitin, which prevents water absorption [41,46].

The trend of the thickness is similar to that of water absorption. The thickness of the swelling of the neat PLA did not significantly change due to its hydrophobic nature. The thickness of the PLA's swelling increased with starch and chitin's addition, as observed in the biocomposite. Generally, this study's swelling thickness depends on the polymer mix's nature and the porosity (voids or space) between the polymer mix molecules [41]. The value of the percentage thickness of swelling is relatively low, which showed that the biocomposite has low porosity and is highly resistant to water [41].

The properties of the biocomposite were further analysed using contact angle analysis as shown in table 3. The contact angle result was observed to reduce with addition of starch and chitin compared with PLA. PLA had the highest contact angle of 85.6° followed with PLA/Mchitin/starch, 79.8°, PLA/chitin/starch, 74.26°, and PLA/starch 68.6°. The contact angle value of PLA/Mchitin/starch had a higher value than PLA/chitin/starch probably due to the acylation substitution which makes the chitin more hydrophobic, while the PLA/starch had the lowest among the biocomposites due to the presence of hydrophilic starch. However, the value of the contact angle showed that all the samples are highly hydrophobic due to higher percentage of PLA content.

**Table 3.** Contact angles of neat PLA and biocomposites.

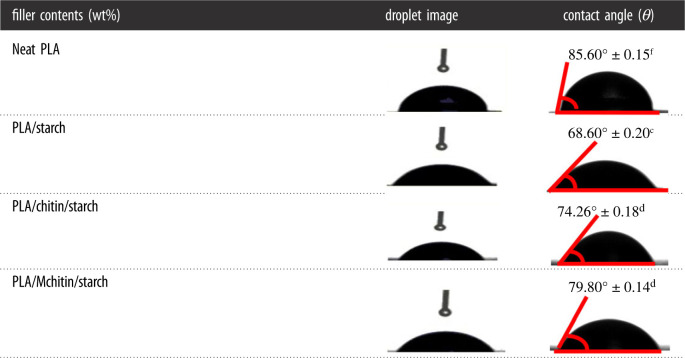

## 4. Conclusion

The biocomposites were successfully developed. The brittleness of neat PLA was reduced with the addition of the blend for all samples. The composites' DMA revealed a strong storage modulus for rigidity and a low loss factor for good miscibility. It also displayed a single peak, indicating that the polymer blend was compatible. The glass temperature from the loss factor peak, on the other hand, indicated that the material was appropriate for industrial use. The thermogravimetry research reveals that the composites have a single deterioration curve and glass transition temperature, indicating stability thermally. The composite moulded with a Carver press demonstrated a homogeneous dispersion of the natural polymer blend in terms of physical appearance. The network distribution of the chitin/starch blend in PLA was seen in SEM images. The morphological features of the blend with no void reveal good compatibility and finely structured network distribution. The photos revealed a uniform combination of modified chitin/starch and PLA compared with other samples.

## Data Availability

The datasets supporting this article can be found in the Dryad Digital Repository at https://doi.org/10.5061/dryad.ffbg79cvw [47].
